# The crystal structure of the *Borrelia burgdorferi* nicotinamidase BBE22 resolves a long‐standing annotation error

**DOI:** 10.1002/2211-5463.70268

**Published:** 2026-05-11

**Authors:** Kalvis Brangulis

**Affiliations:** ^1^ Latvian Biomedical Research and Study Centre Riga Latvia

**Keywords:** borreliosis, Lyme disease, nicotinamidase, PncA, X‐ray crystallography

## Abstract

Nicotinamidases (PncA) catalyze the hydrolysis of nicotinamide to nicotinic acid, a key step in NAD^+^ salvage pathways. In the Lyme disease spirochete *Borrelia burgdorferi*, the plasmid‐encoded gene *bbe22* encodes a PncA enzyme that is essential for survival in both mammalian and tick hosts. Previous genetic and biochemical studies demonstrated that translation of *B. burgdorferi* PncA initiates from a rare non‐canonical AUU start codon, resulting in a protein that is 24 residues longer than the sequence currently annotated in major databases. Despite these findings, public resources such as UniProt and KEGG still list a truncated protein beginning at residue 36, which lacks part of the N‐terminal region required for enzymatic activity. Here we report the crystal structure of full‐length *B. burgdorferi* PncA determined at 3.2 Å resolution. The structure reveals the canonical fold of bacterial nicotinamidases and clear electron density for a ligand in the active site consistent with nicotinic acid, the product of the enzymatic reaction. Structural comparison with homologous PncA enzymes demonstrates conservation of the catalytic architecture, including residues involved in substrate binding and catalysis. Importantly, the experimentally determined structure confirms that the longer N‐terminal sequence described previously is required for formation of the correct fold and active‐site geometry, whereas the truncated annotation is structurally inconsistent with the observed fold and with AlphaFold predictions. Our results provide the first structural characterization of *B. burgdorferi* PncA and resolve the long‐standing annotation discrepancy for *bbe22*, validating the correct protein sequence and providing the structural basis for nicotinamidase activity in this essential metabolic enzyme.

## Graphical abstract

The crystal structure of *Borrelia burgdorferi* nicotinamidase (PncA/BBE22) reveals the correct full‐length protein initiated from a non‐canonical AUU start codon. The structure validates previous biochemical findings and resolves a long‐standing annotation error, demonstrating that the truncated database sequence is structurally incompatible with the active enzyme.

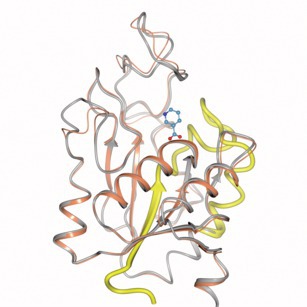

**Abbreviations**
AFDB: AlphaFold Protein Structure Database
NAD^+^
: nicotinamide adenine dinucleotide
PncA: nicotinamidase
RMSD: root‐mean‐square deviation

The spirochete *Borrelia burgdorferi*, the causative agent of Lyme disease, possesses a highly unusual genome composed of a linear chromosome and numerous linear and circular plasmids that encode many genes required for survival during the enzootic cycle between ticks and vertebrate hosts [1, 2]. Among these plasmid‐encoded genes, BBE22, located on linear plasmid lp25, has been shown to be essential for infectivity and survival of *B. burgdorferi* in mammals [3]. Genetic studies demonstrated that loss of lp25 abolishes infectivity, whereas introduction of BBE22 alone restores the ability of the spirochete to infect mice [3]. BBE22 encodes a nicotinamidase (PncA), an enzyme that catalyzes the hydrolysis of nicotinamide to nicotinic acid, a key step in the NAD^+^ salvage pathway [3]. This pathway is particularly important for *B. burgdorferi*, which lacks several enzymes required for *de novo* NAD^+^ biosynthesis and therefore relies heavily on salvage pathways to maintain NAD^+^ homeostasis [1]. Consistent with this metabolic dependence, disruption of *pncA* leads to loss of infectivity in mammalian hosts and impaired survival in ticks. Despite the functional importance of PncA in *B. burgdorferi*, the annotation of the *pncA* gene has long been problematic. Early genome annotations predicted a protein lacking the conserved N‐terminal aspartate residue that participates in the catalytic triad of characterized bacterial nicotinamidases [1, 3, 4]. Subsequent biochemical and genetic analyses demonstrated that translation actually initiates from a rare non‐canonical AUU start codon, producing a protein that is approximately 24 amino acids longer than the originally annotated sequence. This extended N‐terminal region restores the conserved catalytic architecture characteristic of functional nicotinamidases [4]. Nevertheless, major sequence databases such as UniProt and KEGG still list the truncated version of the protein, leading to persistent discrepancies between database annotations and experimentally validated sequence information.

Structural characterization of *B. burgdorferi* PncA has remained lacking. Structures of nicotinamidases from other bacteria, including *Streptococcus pneumoniae* and *Bacillus subtilis*, reveal a conserved fold and catalytic mechanism involving a metal‐assisted hydrolysis reaction that converts nicotinamide into nicotinic acid. Determining the structure of *B. burgdorferi* PncA is therefore important not only for understanding the molecular basis of NAD^+^ metabolism in this pathogen but also for resolving the correct protein annotation and validating the experimentally determined N‐terminal extension.

Here we report the crystal structure of full‐length *B. burgdorferi* PncA corresponding to residues 6–196 of the 202‐residue protein, determined at 3.2 Å resolution. The structure reveals the conserved architecture of bacterial nicotinamidases and contains electron density consistent with nicotinic acid bound in the active site and a coordinated metal. Structural comparisons with homologous enzymes confirm that the extended N‐terminal region is integral to the correct fold and catalytic configuration of the enzyme, thereby providing structural evidence that the truncated annotation present in several databases is incorrect.

## Methods

### Cloning and protein expression

The coding sequence of *B. burgdorferi* BBE22 (UniProt: O50718), corresponding to the full‐length protein (residues 1–202), was synthesized by BioCat GmbH (Heidelberg, Germany). The gene was PCR‐amplified from the synthetic construct and ligated into the pETm‐11 expression vector encoding an N‐terminal 6xHis tag and TEV protease cleavage site. The ligated vector was transformed into *Escherichia coli* XL‐1 Blue cells for selection of colonies, isolation of plasmid DNA, and verification by PCR. The protein was expressed as described previously for *B. burgdorferi* PFam12 member proteins [5].

### Purification of BBE22

The supernatant obtained after *E. coli* cell lysis by sonication followed by centrifugation at 10000 × ***g*** for 30 min, was applied onto Ni‐NTA agarose (Qiagen) column. The bound 6xHis tagged recombinant proteins were eluted from the column with 250 mm NaCl, 50 mm NaH_2_PO_4_, and 300 mm imidazole solution, pH 7.5. To remove the N‐terminal 6×His tag the recombinant protein was mixed with the TEV protease and incubated overnight at room temperature. A second round of affinity chromatography was performed to remove the cleaved 6×His tag and TEV protease. The purified and cleaved protein solution was buffer exchanged into 10 mm Tris/HCl (pH 8.0) and 20 mm NaCl and concentrated to 6.1 mg·mL^−1^ for BBE22 using an Amicon centrifugal filter unit (Millipore).

### Crystallization of BBE22

The purified protein (6.1 mg·mL^−1^ in 10 mm Tris–HCl pH 8.0 and 20 mm NaCl) was mixed with 100 mm nicotinamide stock solution to reach the final concentration of 5 mm, and the complex was crystallized in a sitting drop 96‐well plates prepared using the Tecan Freedom EVO100 workstation (Tecan Group). Initial trials were set up with crystallization screens JCSG‐*plus* and Structure screen 1 & 2 (Molecular Dimensions) followed by optimization. The crystals used for data collection were grown by mixing equal volumes of protein solution and precipitant, containing 25% PEG 3350 and 0.1 M Bis‐Tris (pH 5.5). The crystals were harvested and flash‐frozen by adding 10% glycerol for cryoprotection and stored in liquid nitrogen.

### Diffraction data and structure determination

X‐ray diffraction data were collected at beamline I03 at the Diamond Light Source (Oxfordshire, UK). Diffraction images were processed and indexed using XDS and integrated intensities were scaled and merged using AIMLESS from the CCP4 suite [6, 7]. Initial phases were obtained by molecular replacement using Phaser [8]. An AlphaFold‐predicted model was used as the search model. Automated model building was performed using BUCCANEER [9], followed by iterative manual model adjustment in COOT [10]. The solvent content in the unit cell was determined by MATTHEWS [11]. Crystallographic refinement was performed with REFMAC5 [12]. A summary of data collection, refinement, and validation statistics is given in Table 1. The resolution cutoff at 3.2 Å was selected based on CC1/2, I/σ(I), and completeness criteria, as follows: inclusion of higher‐resolution data did not improve map quality. Bond angle outliers are primarily located in regions of weak electron density, particularly in flexible loop regions, and are consistent with refinement at this resolution.

**Table 1:** Statistics for data and structure quality.

Dataset	BBE22
PDB entry	9TRP
Beamline	Diamond beamline I03
Space group	P 212121
**Unit cell dimensions**	
a (Å)	36.34
b (Å)	45.21
c (Å)	115.21
Wavelength (Å)	0.9762
Resolution (Å)	115.21–3.20
Highest resolution bin (Å)	3.42–3.20
No. of reflections	41 897 (7632)
No. of unique reflections	3391 (603)
Completeness (%)	97.8 (100.0)
R_merge_	0.19 (0.42)
*I/σ* (*I*)	10.2 (5.4)
Multiplicity	12.4 (12.7)
**Refinement**	
R_work_	0.204 (0.249)
R_free_	0.262 (0.491)
**Average B‐factor (Å^2^)**	
Overall	29.7
From Wilson plot	15.6
**No. of atoms**	
Protein	1549
Water	0
**RMS deviations from ideal**	
Bond lengths (Å)	0.007
Bond angles (^o^)	1.567
**Ramachandran outliers (%)**	
Residues in most favored regions (%)	86.91
Residues in allowed regions (%)	13.09
Outliers (%)	0.00

Values in parentheses are for the highest resolution bin.

## Results and Discussion

### Overall structure of *Borrelia burgdorferi* PncA

**Fig. 1:**
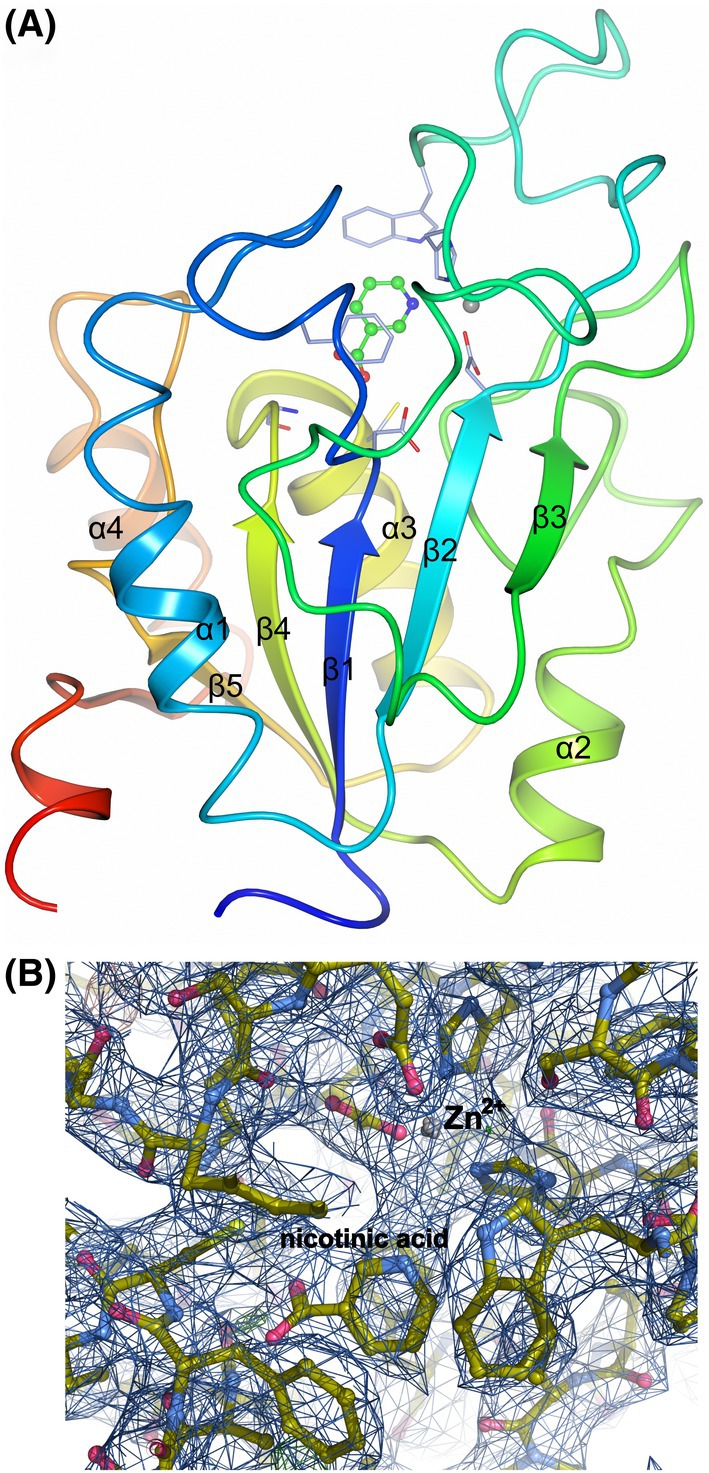
(A) Crystal structure of *B. burgdorferi* BBE22 colored from blue at the N terminus to red at the C terminus. β‐strands (β1‐β5) and α‐helices (α1‐α4) are indicated. Nicotinic acid (stick representation) and the catalytic zinc ion (gray sphere) are shown in the active site, along with selected residues involved in ligand coordination. (B) The 2Fo‐Fc electron density map contoured at 1σ, showing nicotinic acid and a zinc ion in the active site of BBE22.

The crystal structure of *B. burgdorferi* PncA was determined at 3.2 Å resolution using X‐ray crystallography. The refined model of BBE22 comprises residues 6–196 of the full‐length 202‐residue protein, corresponding to the experimentally validated full‐length sequence described previously [4]. The N‐terminal and C‐terminal residues were not modeled due to weak electron density. The overall fold is consistent with that observed for bacterial nicotinamidases and belongs to the conserved hydrolase family that catalyzes the conversion of nicotinamide to nicotinic acid (Fig. 1A). The enzyme adopts the characteristic α/β architecture observed in other PncA homologs, consisting of a central β‐sheet flanked by α‐helices that form the catalytic core of the enzyme. This architecture is highly conserved among bacterial nicotinamidases and is consistent with the enzymatic activity previously demonstrated for BBE22 [3].

### Electron density consistent with nicotinic acid in the active site

Inspection of the electron density maps revealed clear density within the active‐site pocket corresponding to a bound small molecule. The protein was co‐crystallized with nicotinamide; however, the observed density is consistent with nicotinic acid, the product of the nicotinamidase reaction (Fig. 1B), suggesting that enzymatic conversion occurred during crystallization. Modeling nicotinic acid into this density produced an excellent fit without steric clashes and resulted in chemically reasonable interactions with surrounding residues. In addition, electron density consistent with a Zn^2+^ ion was observed in the active site, coordinated by residues Asp52, His54, and His73, in agreement with previously characterized nicotinamidase structures.

### Structural comparison with other nicotinamidases

**Fig. 2:**
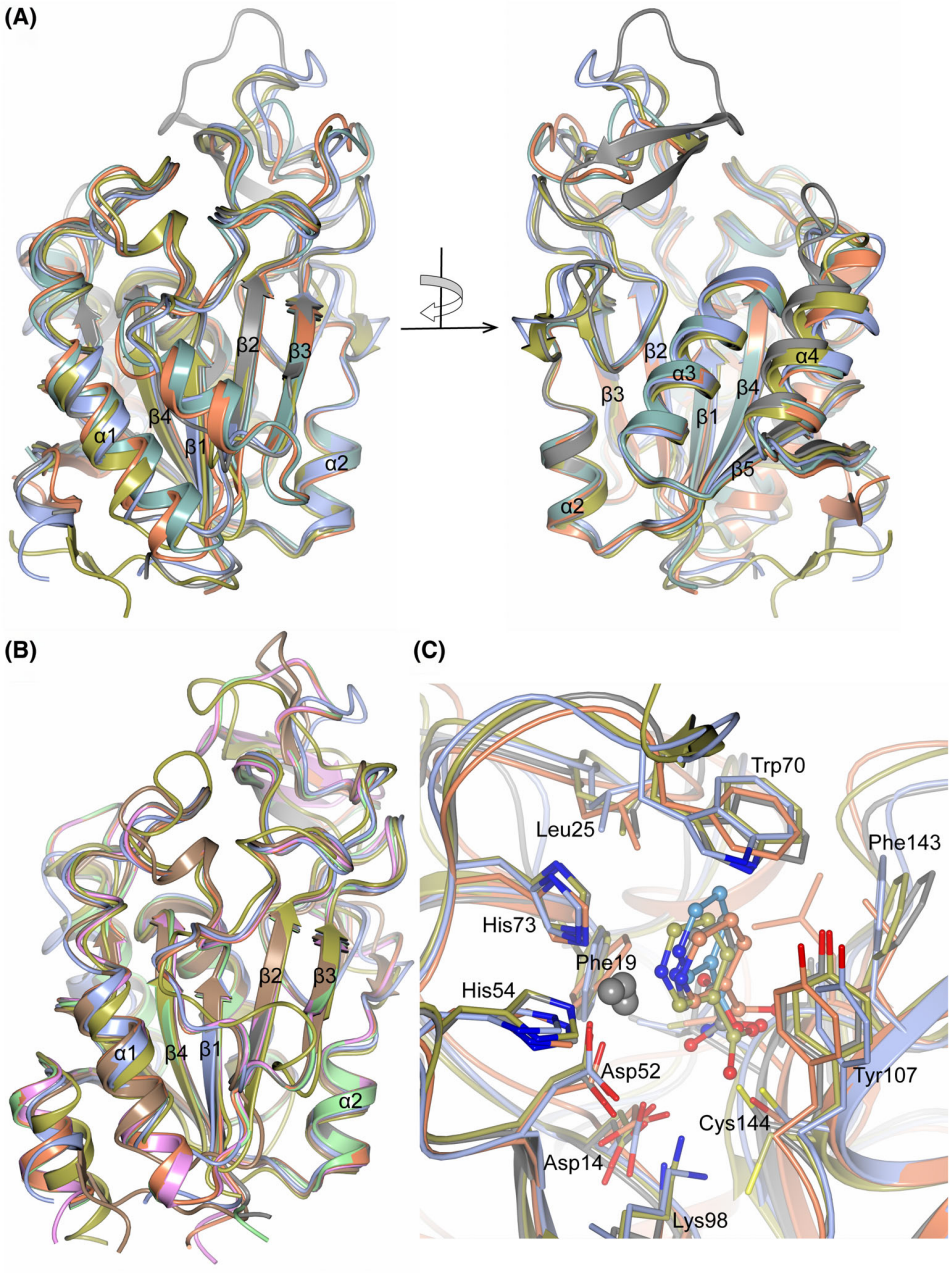
(A) Crystal structure of *B. burgdorferi* BBE22 (residues 6–196, blue) superimposed with PncA from *L. infantum* (PDB ID 3R2J; gold; Cα root‐mean‐square deviation (RMSD) of 1.24 Å), PncA from *S. pneumoniae* (PDB ID 3O94; orange; RMSD of 1.53 Å), PncA from *A. baumannii* (PDB ID 2WT9; gray; RMSD of 1.28 Å), and PncA from *B. subtilis* (PDB ID 5ZN8; green; RMSD of 1.46 Å). (B) Crystal structure of *B. burgdorferi* BBE22 (blue) superimposed with AlphaFold‐predicted PncA structures from *T. brucei* (UniProt: Q38FD5; brown; average pLDDT 96.2; RMSD of 1.11 Å), K. pneumoniae (UniProt: A0A0H3GRH4; green; average pLDDT 96.2; RMSD of 1.22 Å), S. typhimurium (UniProt: Q8ZPV8; pink; average pLDDT 96.3; RMSD of 1.19 Å), S. dysenteriae (UniProt: Q32GB2; gray; average pLDDT 96.3; RMSD of 1.20 Å), E. coli (UniProt: P21369; orange; average pLDDT 97.5; RMSD of 1.18 Å), and N. gonorrhoeae (UniProt: Q5F5J1; gold; average pLDDT 98.0; RMSD of 1.52 Å). (C) The active site of *B. burgdorferi* BBE22 (blue) superimposed with experimentally determined PncA‐nicotinic acid complexes from *L. infantum* (gold), *S. pneumoniae* (orange), and *A. baumannii* (gray). Nicotinic acid and the catalytic metal ion are shown in the active site. Residue numbers correspond to *B. burgdorferi* BBE22.

To assess the structural conservation of *B. burgdorferi* PncA and the binding of the ligand in the active site, the structure was superimposed with previously determined crystal structures of bacterial PncA homologs, including the enzyme from *Leishmania infantum* [13], *Streptococcus pneumoniae* [14], *Acinetobacter baumannii* [15], and *Bacillus subtilis* (Fig. 2A).

Further DALI server [16] analysis against the AlphaFold database (AFDB2) revealed high similarity with AlphaFold [17] predicted PncA structures from various organisms, where the highest similarity was shown for PncA from *Trypanosoma brucei*, Klebsiella pneumoniae, Salmonella typhimurium, Shigella dysenteriae, Escherichia coli, and Neisseria gonorrhoeae (Fig. 2B and Table 2).

**Table 2:** DALI server analysis results for BBE22. The structures correspond to AlphaFold‐predicted models from the AlphaFold Protein Structure Database (AFDB).

	Protein	Organism	UniProt	*Z*‐score	RMSD (Å)	Identity (%)	Length (residues)
1.	Nicotinamidase	*Trypanosoma brucei*	Q38FD5	30.2	1.5	35	204
2.	Nicotinamidase	*Klebsiella pneumoniae*	A0A0H3GRH4	29.2	1.5	34	190
3.	Nicotinamidase	*Salmonella typhimurium*	Q8ZPV8	29.2	1.6	34	218
4.	Nicotinamidase	*Shigella dysenteriae*	Q32GB2	29.1	1.6	36	219
5.	Nicotinamidase	*Escherichia coli*	P21369	29.1	1.5	35	213
6.	Nicotinamidase	*Neisseria gonorrhoeae*	Q5F5J1	26.4	1.7	26	211

**Fig. 3:**
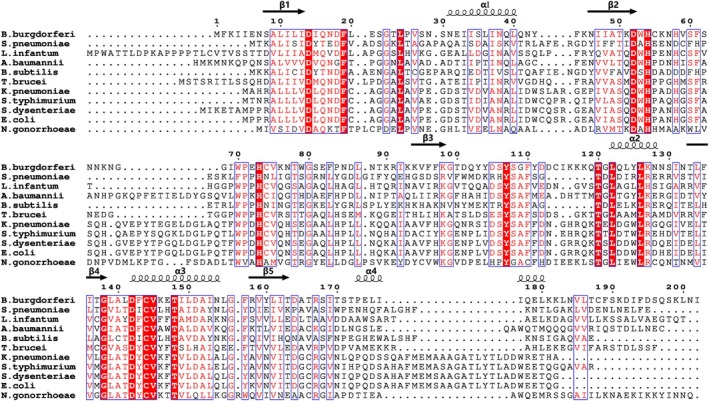
Structure‐based sequence alignment of representative PncA homologs. The alignment was generated using Clustal Omega and visualized with ESPript 3 [18, 19]. Residues strictly conserved among all sequences are shown with a red background, while residues conserved among subsets are shown in red and framed in blue. Secondary structure elements and residue numbering correspond to *B. burgdorferi* BBE22. Key catalytic and substrate‐binding residues are indicated with an asterisk.

The active site is located within a pocket formed by loops connecting the central β‐sheet to surrounding helices. Residues Asp14, Phe19, Leu25, Trp70, Lys98, Tyr107, Phe143, and Cys144 line the substrate‐binding pocket, while Asp52, His54, and His73 coordinate the catalytic metal ion. Structural superposition with PncA structures from *L. infantum*, *S. pneumoniae*, and *A. baumannii* reveals a high degree of conservation within the catalytic core and substrate‐binding pocket (Fig. 2C). The key residues align closely, indicating that the catalytic mechanism is conserved across species. Furthermore, the position of the modeled nicotinic acid in *B. burgdorferi* PncA corresponds well with the ligand positions observed in these experimentally determined complexes. These structural observations are consistent with previous biochemical studies demonstrating that BBE22 possesses nicotinamidase activity and can complement *Salmonella* mutants deficient in NAD biosynthesis [3]. Consistent with these findings, a structure‐based sequence alignment highlights the conservation of key catalytic and substrate‐binding residues across PncA homologs (Fig. 3).

### Structural evidence for the correct N‐terminal annotation

**Fig. 4:**
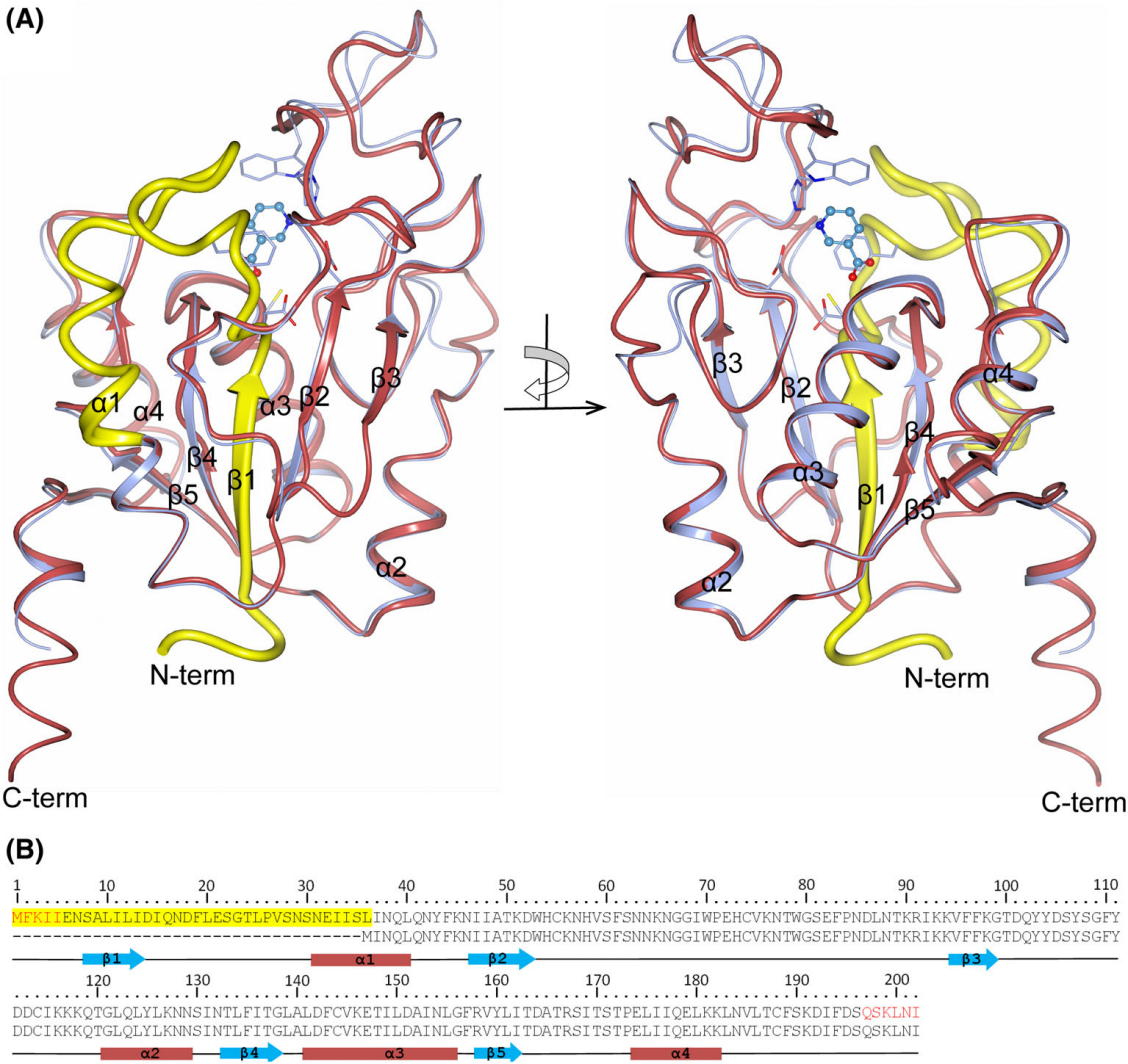
(A) Crystal structure of BBE22 (residues 6–196, blue) superimposed with the truncated AlphaFold prediction from UniProt (red; RMSD 0.67 Å). The N‐terminal segment absent in the truncated model is colored in yellow in the crystal structure. The N‐ and C‐termini are indicated. (B) Sequence alignment of full‐length BBE22 (top sequence) with the BBE22 sequence annotated in the UniProt and KEGG databases. The additional N‐terminal residues present in the full‐length protein are highlighted with a yellow background. N‐terminal and C‐terminal residues not observed in the crystal structure due to weak electron density are shown in red.

One of the key motivations for determining the structure of *B. burgdorferi* PncA was to resolve the discrepancy between experimentally validated protein sequences and database annotations. Earlier studies demonstrated that the *pncA* gene is translated from a rare AUU start codon, resulting in a protein that is longer than the annotated sequence [4]. However, the truncated annotation remains present in several public databases, for example, UniProt (entry O50718) and KEGG for *B. burgdorferi* entry BBE22, including the available AlphaFold prediction within the UniProt of a truncated *B. burgdorferi* PncA. Comparison with AlphaFold predictions based on the truncated database sequence further highlights this discrepancy: models generated from the truncated sequence fail to reproduce the experimentally observed fold, whereas the structure determined here aligns well with predictions based on the full‐length sequence (Fig. 4A).

The crystal structure clearly supports the longer protein sequence. The N‐terminal region contributes to the formation of the correct structural framework that stabilizes the catalytic pocket. This provides a structural explanation for previous biochemical observations that the extended N terminus is required for enzymatic activity [4]. Removal of this region, as implied by the truncated annotation, would disrupt the overall fold and eliminate residues required for proper positioning of catalytic elements (Fig. 4B).

### Implications for annotation of the BBE22 gene

Together, these structural observations provide direct evidence that the currently annotated truncated sequence is incorrect and that the functional PncA enzyme in *B. burgdorferi* corresponds to the longer form initiated from the AUU codon described previously [4].

Given the essential role of PncA in the infectious cycle of *B. burgdorferi*, accurate annotation of this protein is important for studies of bacterial metabolism and for potential therapeutic targeting of NAD salvage pathways in Lyme disease. These results provide the first structural characterization of BBE22, validate the correct sequence, and establish the structural framework of *B. burgdorferi* PncA, resolving a long‐standing annotation discrepancy in major protein databases.

## Conflict of interest

The author declares no conflict of interest.

## Author contributions

KB designed and planned the study, performed the experiments, analyzed and interpreted the data, acquired funding, and wrote the manuscript.

## Data Availability

The coordinates and structure factors for *B. burgdorferi* BBE22 have been deposited in the Protein Data Bank with the accession number 29IV.
